# Mapping Oil Spills from Dual-Polarized SAR Images Using an Artificial Neural Network: Application to Oil Spill in the Kerch Strait in November 2007

**DOI:** 10.3390/s18072237

**Published:** 2018-07-11

**Authors:** Daeseong Kim, Hyung-Sup Jung

**Affiliations:** Department of Geoinformatics, University of Seoul, Seoul 02504, Korea; kds2991@uos.ac.kr

**Keywords:** artificial neural network (ANN), co-polarized phase difference (CPD), non-local means filter (NL-means filter), oil spill detection, probability map

## Abstract

Synthetic aperture radar (SAR) has been widely used to detect oil-spill areas through the backscattering intensity difference between oil and background pixels. However, since the signal is similar to that produced by other phenomena, positive identification can be challenging. In this study we developed an algorithm to effectively analyze large-scale oil spill areas in SAR images by focusing on optimizing the input layer to artificial neural network (ANN) through removal the factor of lowering the accuracy. An ANN algorithm was used to generate probability maps of oil spills. Highly accurate pixel-based data processing was conducted through false or un-detection element reduction by normalizing the image or applying a non-local (NL) means filter and median filter to the input neurons for ANN. In addition, the standard deviation of co-polarized phase difference (CPD) was used to reduce false detection from the look-alike with weak damping effect. The algorithm was validated using TerraSAR-X images of an oil spill caused by stranded oil tanker Volganefti-139 in the Kerch Strait in 2007. According to the validation results of the receiver operating characteristic (ROC) curve, the oil spill was detected with an accuracy of about 95.19% and un-detection or false detection by look-alike and speckle noise was greatly reduced.

## 1. Introduction

Marine oil spill disasters are one of the major concerns in the ocean environment [1]. Oil spills can be caused by people making mistakes, equipment breaking down, natural disasters, etc. The 2010 Gulf oil spill and the 2011 Bohai bay oil spill are well-known large marine oil spill events [2,3,4,5]. Oil rapidly spreads out on the water’s surface, and it forms a thin oil slick. As the oil continues spreading, the oil slick gets thinner, and consequently the slick becomes a very thin sheen. The oil spills can be very harmful to birds, turtles, fishes and shellfish. Thus, it has a negative effect on both economy and ecosystem. The economic damage is caused by the destruction of the ecosystem, and then the oil spill pollution causes, not only immediate damage, but also persistent problems [6]. In order to minimize the damage, it is most important to monitor the oil spill and establish proper measures according to the monitoring results.

It is well-known that oil-covered pixels appear dark in synthetic aperture radar (SAR) images because oil spills on the ocean surface dampen Bragg scattering [7,8,9]. Based on the characteristics, various studies have focused on the detection of oil spills. The most commonly used method for oil spill detection is to divide the image into two parts by applying the threshold value which is determined in a bimodal histogram created by oil-free surface and oil-covered surface. Since it is simple and accurate, many studies have proposed an oil spill detection algorithm based on the simple or adaptive thresholding techniques [10,11,12,13]. Further improved oil spill detection algorithms, which are based on the intensity difference between the target and background pixels, have been proposed by exploiting (i) the texture information of sea surface cover types [14,15,16,17]; (ii) the statistical value of oil spills [17,18,19] or (iii) the edge detection between oil spill and the oil-free surface [20,21,22,23]. However, the methods suffer from a false detection problem due to look-alikes that produce a similar signal with oil spill; for example, low-wind zones (wind speed < 3 m/s), internal wave, ship wake and biogenic films [24,25]. To overcome this issue, morphological features of oil spills (i.e., perimeter, area, and complexity) or wind data have been used for oil spill detection [13,26]. However, it can be difficult (i) to define standard shapes for oil spills owing to the high fluidity or (ii) to distinguish the shape of oil spills from that of look-alikes (e.g., ship wake and biogenic films).

Several methods using machine learning have also been proposed [3,26,27,28,29,30,31]. In most studies, an artificial neural network (ANN) of object-based algorithm is usually applied to detect oil spills, the first reason is to use the morphological features as input neurons. The second is that the machine learning method is highly dependent on the input layer, especially, there is a problem that false detection and non-detection due to the noise of the input data is greatly increased when using the pixel-based ANN algorithm. Thus, most studies have adopted an object-based algorithm. However, the object-based algorithm has problems that the dark spot detection accuracy is considerably low, and it is furthermore difficult to automate the algorithm. Moreover, in the case of massive outflow accidents (e.g., the sinking of an oil tanker), it is almost impossible to apply the algorithm because oil forms one big object and normal morphological characteristics can be significantly altered.

Recently, studies have been conducted to improve the accuracy of detection by using the polarization information. Four polarized images (HH, HV, VV, and VH) can be obtained from SAR sensors according to the transmitting and receiving modes of the radar signal. Among them, co-polarization (HH or VV) occurs when transmit and receive polarizations are identical. The phase difference between VV- and HH-polarized images can be represented by the co-polarized phase difference (CPD), and CPD has a higher correlation with oil spill than it does with look-alikes, which have a weak-damping effect [32]. In addition, the coherence between VV- and HH-polarized images are very low in oil spill areas, whereas it is very high in oil-free areas, which is close to 1. The coherence denotes the degree of correlation between two complex signals. For more optimal solution of oil spill detection using the co-polarized information, we need to focus on the creation process of optimal input neurons for the ANN algorithm from co-polarized SAR imagery, because the creation of optimal input neurons is very important in the ANN technique [33].

In this study, we propose an efficient method to identify oil spill areas from co-polarized TerraSAR-X imagery using the artificial neural network (ANN) and validate it using the oil spill event in the Kerch Strait in November 2007. For this, we focus on (i) the oil spill enhancement from the intensity and phase information of the co-polarized TerraSAR-X imagery and (ii) the precise identification of oil-covered surface from the oil spill-enhanced images via the ANN approach. Four maps were extracted from the HH and VV single-look complex (SLC) images, which include the intensity, texture, coherence and phase texture maps, and then oil-covered areas in the maps were emphasized through multi-looking and the non-local (NL) means and median filtering. The ANN approach is used to generate an oil spill probability map from the four emphasized maps, and then oil-covered areas are identified from the probability map. The oil spill detection performance is validated by using the receiver operating characteristic (ROC) curve. In addition, we compare between the results from the presence and absence of the phase information, because most SAR images are acquired from the single-polarization. The proposed method has the advantage that it can estimate the oil spill probability at the pixel unit, and hence it would be effectively used for large-scale oil spill accidents. Moreover, the proposed method enables us (i) to reduce false detection associated with look-alike as well as (ii) to improve the detection performance of oil spill areas.

## 2. Data and Study Area

**Figure 1:**
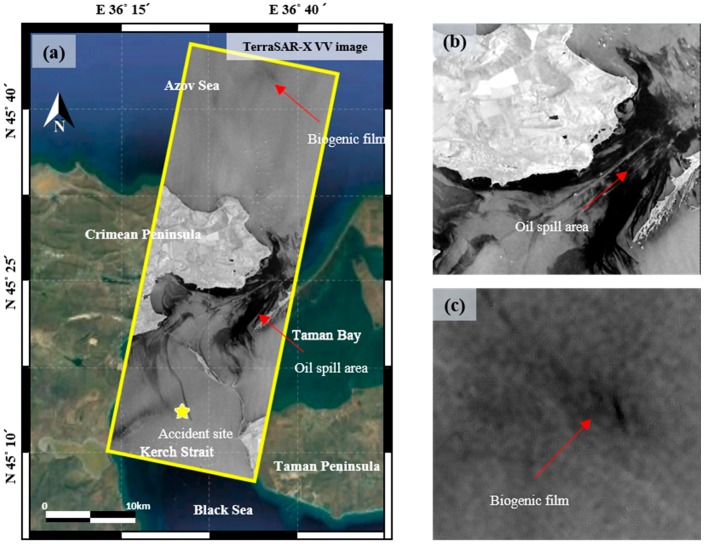
(**a**) Kerch Strait study area shown in the TerraSAR-X image acquired on 16 November 2007. The star denotes the location of the Volganeft-139 accident. (**b**) Magnified image of the oil spill area. (**c**) Magnified image of a biogenic film region.

The X-band TerraSAR-X SAR data with the HH and VV dual polarization of the standard mode was used in this study, as shown in Figure 1a. The HH and VV images were provided in the single look complex (SLC) format, and they were obtained in five days after an oil spill accident on the descending pass with the incidence angle of about 26 degrees. It has been reported that the low backscattering areas in the ocean clearly show oil-spill accident areas [32,34]. Table 1 summarizes the imaging characteristics of the test image used for this study. As seen in Figure 1a, it is very clear that oil-covered surface is very dark in the TerraSAR-X image even when compared with sea surface. The characteristics of the SAR image enable us to detect oil spill areas easily. Nevertheless, false detection problems due to speckle noise and look-alikes are present, as aforementioned. The 30-m shuttle radar topography mission (SRTM) digital elevation model (DEM) was used to mask land area in order to remove false alarms from the land area.

On 11 November 2007, a large storm occurred in the Black Sea, causing winds in excess of 35 m/s and waves of over 5 m in height. The storm resulted in four vessel accidents, including the oil tanker Volganeft-139, which became stranded and within four days had spilled 2000 tons of oil into the sea. The oil impacted on both the Taman and Crimean peninsulas depending on wind and ocean currents [35]. When the SAR image was obtained on 16 November 2007, the wave height and wind speed are about 2 m and 10 m/s, respectively [34]. The ocean currents in the Kerch Strait during the period of 11–16 November were exclusively directed from the Black to the Azov Sea with branches from the Kerch Strait to the Taman Bay [34]. More details of the wind condition and ocean currents can be found in [34,35]. The location of sunk of Volganefti-139 is shown in Figure 1a. The oil spill area became widely distributed as seen in Figure 1b, and a region of look-alike is visible, as shown in Figure 1c.

## 3. Data Processing

Generally, the two types of information that can be acquired from SAR images are intensity and phase. The intensity gives us the reflectivity information of the surface roughness and dielectric constant, while the phase enables us to estimate the scattering information of the object. Since the intensity and phase information are largely dependent on objects, they are allowed to identify the objects from the information. Moreover, texture information can be additionally obtained from both the intensity and phase images. The texture information can be used to further improve object identifications.

**Figure 2:**
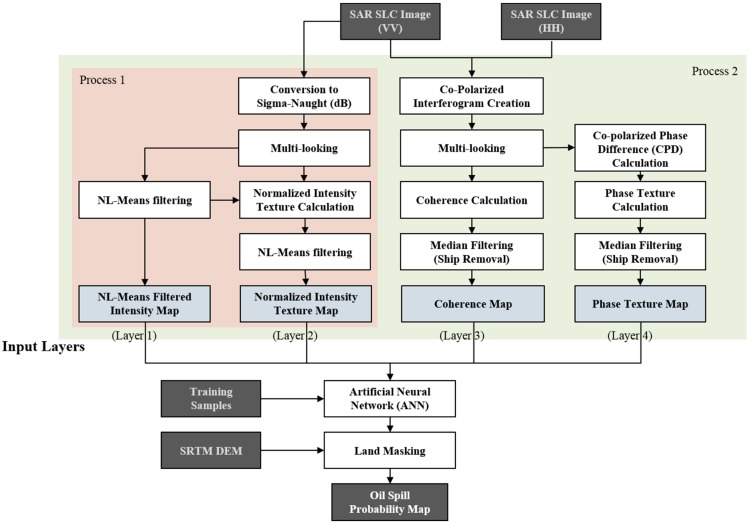
Flow chart of proposed algorithm.

Figure 2 is a detailed workflow of data processing designed for this study. The HH and VV SLC images were used for generate four input neurons, which are composed of (i) NL-means filtered intensity map; (ii) normalized intensity texture map; (iii) coherence map, and (iv) phase texture map. The ANN was learned by using pixel values of training samples from the four input neurons and an oil spill probability map was then generated by processing through learned neural network and land masking data. The most important points when creating the input layer are (i) to enhance the contrast between target and background pixel values and (ii) to eliminate elements that can cause false detection or un-detection. In the process of generating each input neuron, filter techniques were applied to remove factors that degraded accuracy. In the intensity images, an NL-means filter was used to remove speckle noise that caused un-detection and false detection. In the phase texture and the coherence maps, a median filter was applied to remove ships, which were false sensing elements. In the learning steps of the neural network, a back-propagation algorithm was used. This algorithm updates the weights of hidden layers to minimize total error at the output layer. The ANN algorithm implemented in MATLAB software was used in this study. Four input neurons were learned with sigmoid function and neural networks were iteratively performed during 2000 epochs with 0.01 learning rate. Consequently, eight neurons were in the hidden layer and one linear output layer was created. The hyperparameters were determined by an empirical evaluation. In this study, oil spill samples of ten thousand pixels were selected from the oil spill areas reported by [31,33]. A total of 50% of the oil spill samples were randomly selected as training/validating data for the two-fold cross-validation, and the others were determined to be test data. The look-alike pixels were used as the non-oil spill training pixels.

We tested two processes to compare between the results from the presence and absence of the phase information. Process 1 generates the probability map of oil spill only using intensity information of a single-polarization. That is, NL-means filtered intensity map and normalized intensity texture map were used as the input neurons in the process 1. Process 2 utilizes both the HH and VV polarized images and uses phase information together with intensity information. In this case, the NL-means filtered intensity map, normalized intensity texture map, coherence map, phase texture map were used for the process 2. The comparison of classification performances between the two processes was performed by using ROC curves.

### 3.1. Generation of NL-Means Filtered Intensity Map

Speckle noise in SAR images is an inevitable factor in signal processing. The speckle noise can make a pixel value extremely high or low compared with surrounding pixels, and hence it causes un-detection or false detection. The multi-look technique is commonly used to minimize speckle noise, but it degrades the spatial resolution of the image. An adoptive filter can be applied to reduce the speckle noise, but most filters have a trade-off relationship between the spatial resolution and noise removal. Recently the NL-means filter was designed to reduce image noise while minimizing resolution loss. More details can be found in [36]. The NL-mean filtered intensity map is generated by three steps as follows:

(i) sigma-naught image is generated from the SLC VV image,
(ii) multi-looked image is created from the sigma-naught image, and
(iii) NL-means filtered intensity map is created from the multi-looked image.

The first step is applied to make the probability density function (PDF) being a Gaussian, and the second step is used for the speckle reduction. However, since the multi-look process degrades the spatial resolution, small multi-look size is used in both the azimuth and range directions. Further noise reduction is performed by the NL-means filter. Since the HH polarization has a high dependency on incidence angle, so as the angle increases, the intensity value of the sea decreases accordingly. This phenomenon makes the distinction between sea and oil spill more difficult in HH images [37]. For this reason, the VV-polarized image is used for generating the intensity map.

### 3.2. Generation of Normalized Intensity Texture Map 

Since the oil-covered and oil-free surfaces have different textural characteristics, the texture information can be used to distinguish them. The texture information can be simply calculated by using standard deviation. However, since the standard deviation can be biased, the texture information is estimated by the root-mean-square difference (RMSD) between the multi-looked and NL-means filtered images, which are defined from the Section 3.1, and then normalized by the NL-means filtered image. The normalized texture map of the intensity image is constructed by three main steps as follows:

(i) difference image is generated by subtracting the NL-means filtered intensity map from the multi-looked image,
(ii) texture image is produced by the root-mean-square of pixel values of the difference image within the moving window and normalized by pixel value of the NL-means filtered intensity map, and
(iii) normalized intensity texture map is created by the NL-means filtering of the texture image.

The normalized intensity texture is defined by:

(1) $$N\left(x\right)=\frac{\sqrt{\mathrm{sum}\left(M\left[dVV^{2}\left(x\right)\right]\right)}}{T_{VV}\left(x\right)},$$

where N(*x*) denotes the value of normalized intensity texture map, sum(∙) denotes the sum of all matrix elements, *dVV*^2^ is the squares of each element of difference map acquired by subtracting non-filtered VV from the filtered VV intensity image and *T_VV_* is filtered VV intensity image. The NL-means filter is applied to the computed N(*x*) to reduce the noise component and then used as a normalized intensity texture map, which is the second input neuron.

### 3.3. Generation of Normalized Intensity Texture Map 

Coherence is an index of correlation between two different SLC images, in which oil spill and oil-free surfaces have opposite characteristics to those in the phase texture map. The signal from an oil-free ocean surface is recorded according to the Bragg scattering model, with the values of these pixels high. However, in oil spill areas, pixel values are remarkably decreased because the Bragg scattering model is not followed. The coherence image was calculated by the equation [38]:

(2) $$\rho =\frac{\left|E\left[S_{hh}S_{vv}{}^{*}\right]\right|}{\sqrt{E\left[{\left|S_{hh}\right|}^{2}\right]}E\left[{\left|S_{vv}\right|}^{2}\right]},$$

where ρ denotes coherence, *E*[∙] is the ensemble average operator, and *S_hh_* and *S_vv_* denote the SLC images of the HH and VV polarizations, respectively. Coherence places a value between 0 and 1, and a value at near 1 means greater correlation of two images.

For ship pixels, the signal is dominantly returned by a double-scattering model and coherence significantly decreases resulting in a coherence value close to that of the oil-spill area. Therefore, as in the phase texture map, the ship acts as a false detection element. To combat this, the image without the ship was obtained by applying the median filter and uses for input neuron.

### 3.4. Generation of Phase Texture Map

The phase texture map is a standard deviation image of the CPD. When phase information of the VV is subtracted from the phase information of the HH, the distribution of values is almost uniform for look-alike and oil-free ocean surfaces, but is noisy in oil spill areas; therefore, the standard deviation image of CPD has a high value in the oil spill region and a relatively low value in the other pixels, making it possible to reduce the false detection of biogenic film. This can be calculated using the formula [39]:

(3) $$\varphi =∠\left(S_{hh}S_{vv}{}^{*}\right)=\varphi_{hh}-\varphi_{vv},$$

(4) $$\sigma_{\varphi \left(x\right)}=\mathrm{std}\left[M\left(\varphi \left(x\right)\right)\right],$$

where φ(x) is the value of CPD at pixel location *x*; ∠ and * are phase and complex conjugate, respectively; $\varphi_{hh}$ and $\varphi_{vv}$ are phase information obtained by the respective polarizations, $\sigma_{\varphi \left(x\right)}$ is the value of phase texture map at location *x*, and std[·] is the operator that calculates the standard deviation of all matrix elements.

Look-alike areas were eliminated by using the texture map of the phase, but where a pixel value was similar to oil spill, a false detection would occur. It is highly likely that a ship will be detected as an oil spill, so a median filter was used to remove it, producing a phase texture map without a ship.

## 4. Results and Discussion

**Figure 3:**
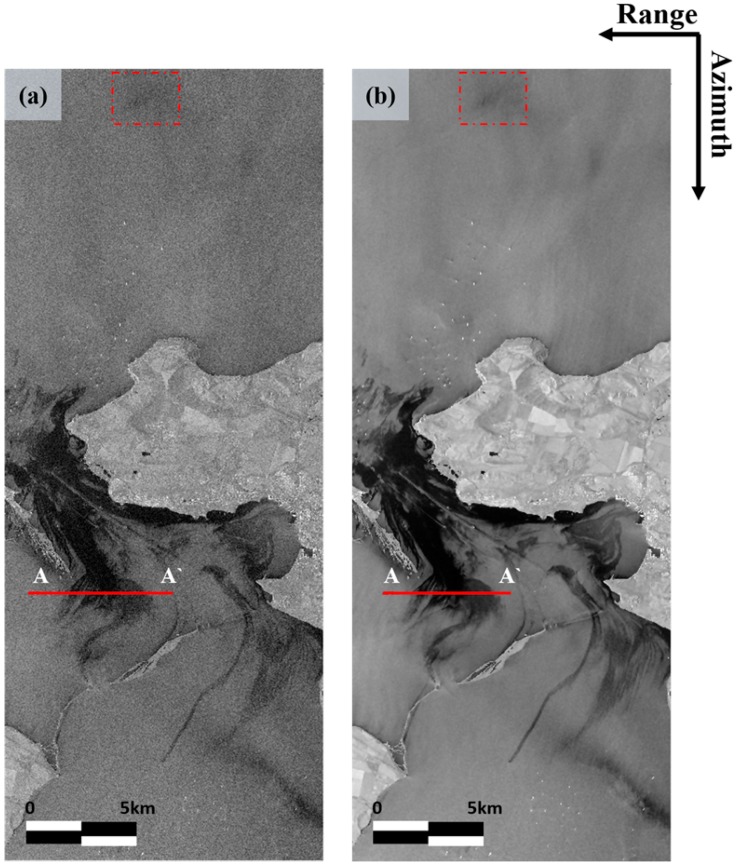
Intensity images before and after applying non-local (NL)-means filter: (**a**) before and (**b**) after filtering.

Figure 3 shows the image before and after applying the NL-means filter to the VV intensity image. Since speckle noise distort the true pixel value, in the image before applying the filter, pixel having a darker value than surrounding pixels could be observed even it is located in the oil-free region. Likewise, some pixel which is significantly bright compared to adjacent pixels could be exists in the oil-covered region (Figure 3a). These cause false detection and un-detection in oil spill detection, respectively. On the other hand, in NL-means filtered image, it can be seen that the same type of cover have substantially similar brightness values to each other without degrade of spatial resolution. That is, no downgrade of image quality was observed in the NL-means filtered image, and the oil spill and oil-free areas were easily distinguished (Figure 3b). However, a biogenic film region checked with red box still had similar pixel values to the oil spill. In order to visually confirm the change of the pixel values before and after the filter, we specified a line (start at the A and end at the A’) for generating the profile map.

**Figure 4:**
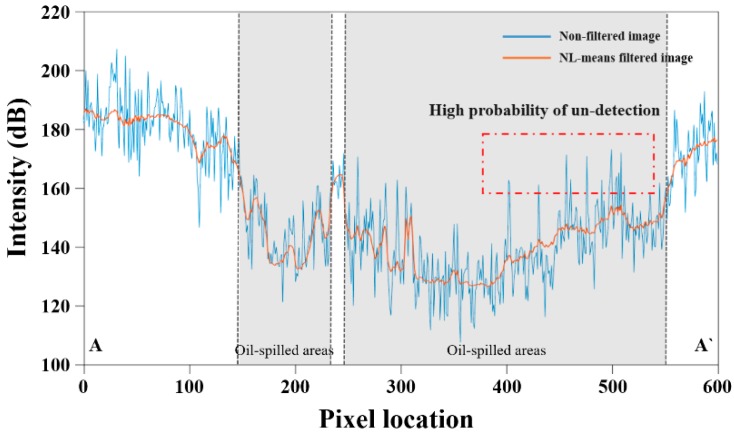
Profile map from A to A’. Points in the red box indicates a pixel that has high possibility of un-detection. Blue color line is generated by value of non-filtered image pixel and value of NL-means filtered image is drawn with orange color line.

Figure 4 shows two profile maps simultaneously. The blue color line is generated using the value of image before applying the NL-means filter and the orange color line is generated using the NL-means filtered image’s value. It can be seen that the blue color line oscillates greatly in the vertical direction, which means that the difference between the adjacent pixels is large due to the speckle noise. In contrast, the orange color line changes relatively smoothly and slightly. The most notable range in the profile map is red dot box area. Some points of blue line is marked high position, even though it is an oil spill area. When the oil spill is detected using the image having these value, there is a high possibility that the pixels in the box are not classified as oil spill. Likewise, in the oil-free region, the blue line points are observed to be relatively lower than the orange line. These pixels are highly likely to be classified as oil spill. Therefore, the value of orange line is more effective in detecting oil spill.

**Figure 5:**
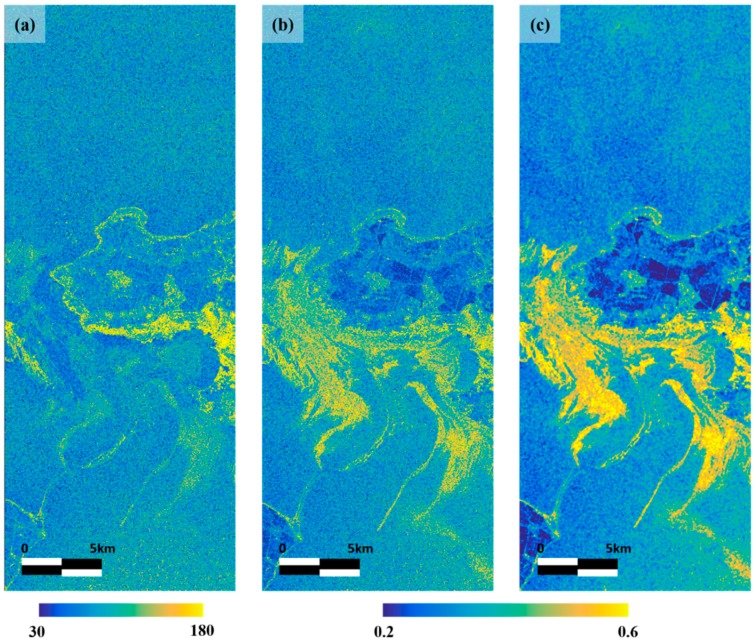
Generation of normalized intensity texture map. (**a**) Texture map is generated by applying the sum of square to difference image which is made by subtracting non-means filtered image from NL-means filtered image; (**b**) Normalized intensity texture map by using NL-means filtered image; (**c**) Generated final map applying NL-means filter.

Figure 5 shows a normalized intensity texture map, which is the second input neuron of ANN. The map just has a correlation with some parts of oil spill, urban area and coast line (Figure 5a). The pixel value of urban area is calculated high because there is variety scatterer, and since the map was calculated using a moving window, values were also high along the coastline. However, after normalization by using NL-means filtered intensity map, the values of urban area and coast line are relatively reduced, while the values of all oil spill areas is entirely increased (Figure 5b). Especially, in the thin oil spill region, there was almost no difference of the value with thick oil spill region. However, since the texture map which is obtained using the sum of squares has high noise level. So, the NL-means filter is applied to degrade the noise of texture map. The normalized intensity texture map showed a high correlation even in the thin oil spill area but the look-alike area also has a similar value to that of oil spill.

**Figure 6:**
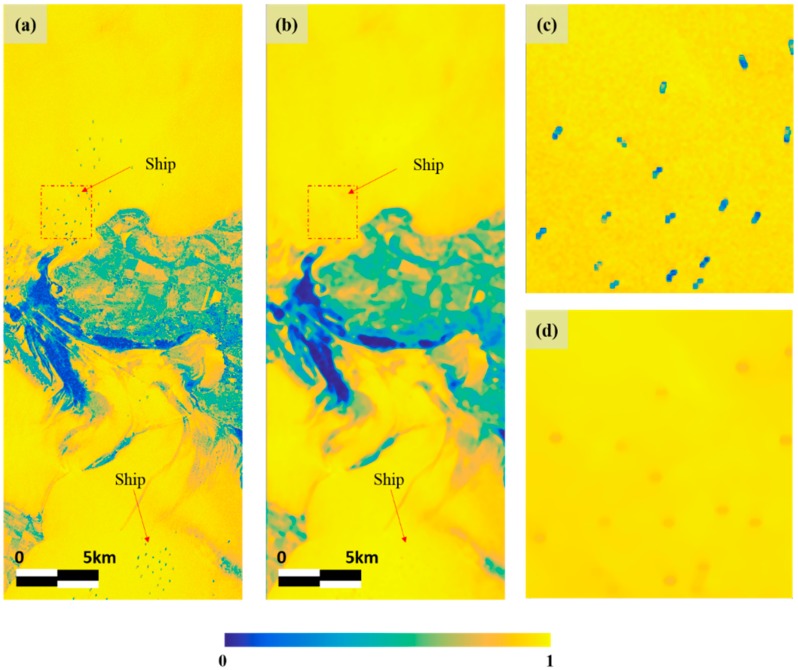
Coherence map before and after ship removal. (**a**) Coherence map before ship removal; (**b**) Coherence map after ship removal; (**c**) Magnified coherence map of ship area before ship removal; (**d**) Magnified coherence map of ship area after ship removal.

The pixels in the same cover type of the coherence image have similar value. In other words, the contrast between ocean and oil spill is so large that oil spills can be effectively detected. Figure 6a shows the coherence image before the ship removal and Figure 6b shows the coherence image after the removal. The value of oil-free surface, as mentioned earlier, is close to 1, and oil spill area and the ship can be seen that a value closer to zero. So, to reduce false detection, the ship must be removed. In this study, the median filter with window size of 21 × 21 was applied to the image for removing the ship. Figure 6c,d is a before and after the filtering of magnified image which includes ship. In filtered image, it can be seen that the value of the ship is much more similar to that of the surrounding ocean, compared to the ship which was clearly contrasted with the sea in image before applying the filter. Even though the ship is not completely removed from the coherence image, there is a very low probability of false detection, because the oil spill area and ship are clearly contrasted in intensity based images which is other input neurons of the ANN approach using coherence map.

**Figure 7:**
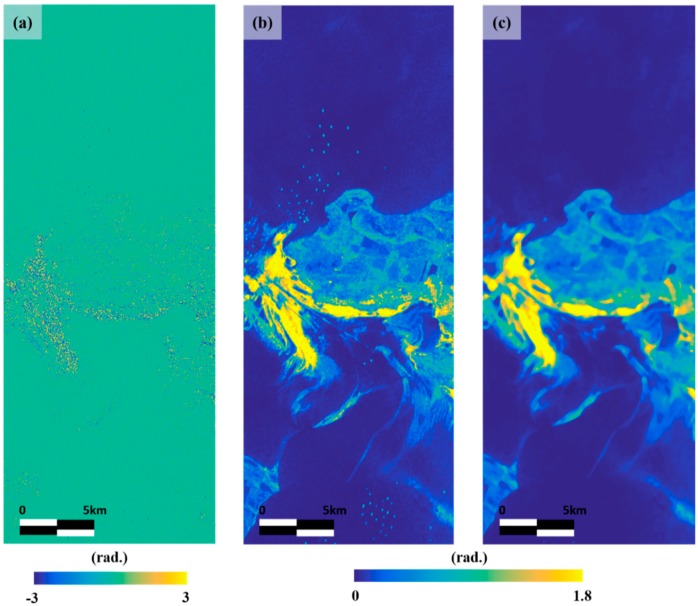
Generation of phase texture map: (**a**) CPD image; (**b**) phase texture map before ship removal, and (**c**) hase texture map after ship removal.

Figure 7a shows the CPD image, which is generated from the HH and VV polarizations image, so that the oil-free surface is generally close to zero, and it is noisy in the land, ship and oil spill areas. Figure 7b shows that the phase texture image obtained by applying Equation (4) to the CPD image. This map has a high correlation with the oil spill and a low value in the look-alike region having the weak-damping effect. In look-alike region, this map contained no bright pixels. Therefore, if using the phase texture image which have been removed ship, it is possible that oil spill is accurately detected without false detection caused by look-alike and ship. As in the case of removing the ship from the coherence image, the median filter was applied to the image and the signal by the ship was reduced (Figure 7c).

**Figure 8:**
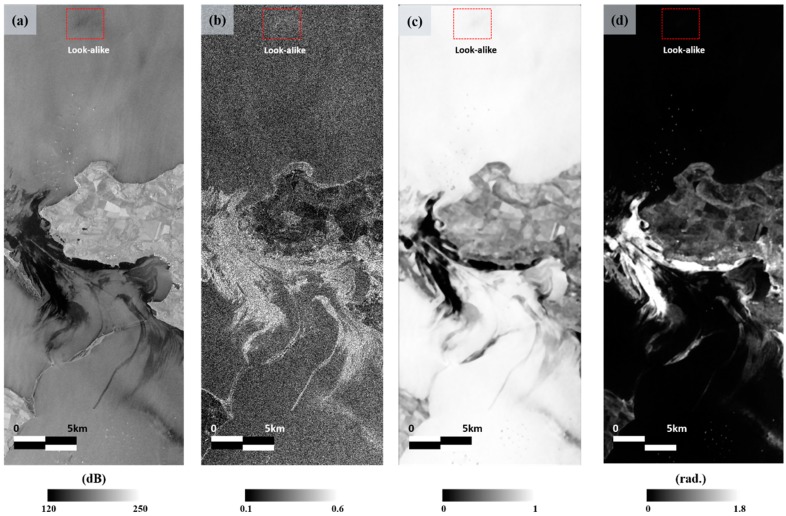
Inputs for the artificial neural network (ANN) with the look-alike region denoted by the red box and the ship pixel marked by a dotted red box: (**a**) NL-means filtered intensity map; (**b**) normalized intensity texture map; (**c**) coherence map; and (**d**) phase texture map.

Figure 8 shows the four input neurons of the ANN. The oil spill and oil-free surfaces are clearly distinguished from each other in the four images. In particular, thick oil is more distinguishable in Figure 8c,d generated from the phase information compared to other images. In addition, in the look-alike region with red box, its value is similar to the surrounding ocean value unlike Figure 8a,b, because it is well-known that the PDF of the look-alikes are very close to that of oil-free surfaces in the coherence and phase texture maps [32]. However, the thin oil areas could be more easily recognized from the images generated by intensity information, and the strong contrast between oil spill and ship was found in Figure 8c,d.

**Figure 9:**
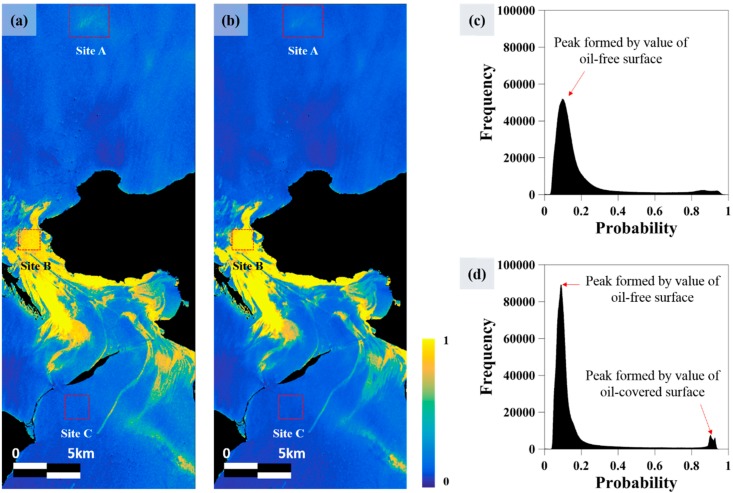
(**a**) Probability map of oil spill estimated following Process 1; (**b**) Probability map of oil spill estimated following Process 2; (**c**) Histogram of probability for the result of Process 1; (**d**) Histogram of probability for the result of Process 2.

Using the four neurons, oil spill probability maps were generated. When generating the probability maps, 50% of the oil spill area (as classified by visual inspection) was used to train and validate the neural network model. Figure 9a,b shows oil spill probability maps estimated by Process 1 and Process 2, respectively. The results of Process 1 and Process 2 show no significant difference when compared it visually. Both results seem to have properly detected the oil spill area. The Figure 9c,d shows the histogram of each probability to compare the distribution of the values. In Process 1, a peak is observed around a probability of 0.1, which is formed by pixels corresponding to the oil-free surface. The frequency of other probabilities except the probability due to the oil-free surface is not much different. In other words, it is difficult to determine the threshold value for distinguishing between oil spill and oil-free because the peak due to oil spill is not observed clearly. On the other hand, in Figure 8d, peaks are observed at a probability of about 0.1 and one peak is additionally observed at around 0.9, which is a pixel representing oil spilled on the ocean. It can be seen that it is easier to determine the threshold between the two covers in probability because the two distributions have distinct peaks. That is, when all four input neurons are used, it means that the oil spill area can be detected more accurately. A (look-alike region), B (oil spill region) and C (oil-free surface region) sites were selected to compare the probabilities of the two results according to cover type.

**Figure 10:**
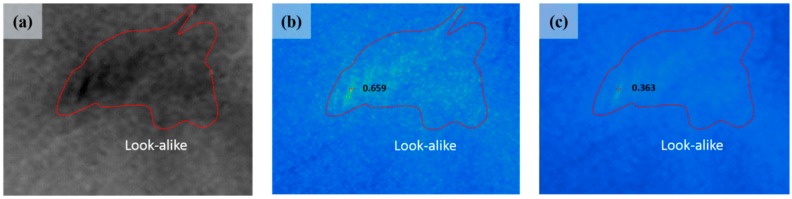
(**a**) Enlarged image of look-alike area in intensity image; (**b**) Enlarged image of look-alike area in Figure 9a; (**c**) Enlarged image of look-alike area in Figure 9b.

Figure 10a–c shows the look-alike region of intensity, and the results of Process 1 and Process 2, respectively. Using our new procedure, the probability of the look-alike was clearly lowered (Figure 10c). For the probability map calculated using only the intensity layers, the highest probability value of a look-alike was ~0.65 (Figure 10b). For the probability map calculated using all layers, the highest probability value of a look-alike was less than ~0.36 (Figure 5c). This reflects the ability of the phase texture map to distinguish biogenic film and oil spills. Based on these results, we confirmed that using dual-polarized HH and VV phase information together is more effective for detecting oil spills than using only intensity images.

**Figure 11:**
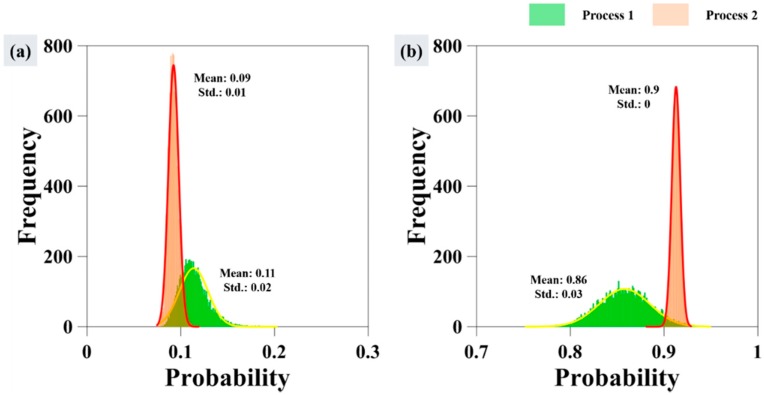
Histogram of sites B and C. (**a**) Histogram of results of Process 1 and Process 2 at oil-free surface; (**b**) Histogram of results of Process 1 and Process 2 at oil-covered surface.

Figure 11a is the histogram of site C and Figure 11b is the histogram of site B. The histograms represented by green color in both graphs are based on Process 1, and the histograms in red color are based on Process 2. In the histogram of Process 1, the probability of an oil-free area being oil spill was relatively higher than in the probability map using phase information together with intensity information. Whereas oil-covered pixel is represented slightly low. As a result of numerical analysis, in process 2 using phase information, mean and standard deviation of oil-free region decreased by 0.02 and 0.01, respectively. In oil region, mean value decreased by 0.04 and standard deviation of 0.03. This difference is due to the coherence and phase texture maps. Both images showed a distinct contrast value between oil spill and oil-free ocean surface pixels. Coherence values were closer to 1 in ocean areas, but considerably smaller in the oil region. In contrast, for the phase texture map high pixel values were seen in the oil spill area, whereas low values were seen for ocean surface areas. So, the probability map produced using the proposed method allow for the clear discrimination.

**Figure 12:**
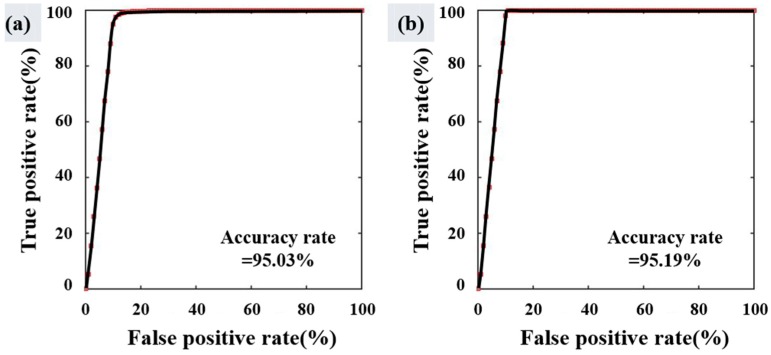
(**a**) Validation of Figure 9a using a receiver operating characteristic (ROC) curve, where the area under curve (AUC) is about 93.63%; (**b**) Validation of Figure 9b using a ROC curve, where the AUC is about 93.77%.

The results were validated using a receiver operating characteristic (ROC) curve where the X-axis represented 1-specificity (where a false case is predicted to be false) and the y-axis represented sensitivity (where a true case is classified to be true; Figure 6). In this study, specificity relates to non-oil spill pixels judged to be oil-free pixels, whereas sensitivity relates to oil spill pixels classified as oil-covered pixels. The area under the curve (AUC) is the calculated value of the bottom area of the ROC curve. AUC values range from 0.5 to 1, and those closer to 1 (100%) represent more meaningful models. Values less than 0.5 are considered to be worthless [40]. According to [41], AUC is divided into five levels: excellent (0.9–1.0), good (0.8–0.9), fair (0.7–0.8), poor (0.6–0.7), and fail (0.5–0.6). In this study, 50% of the oil spill samples was used for the training data and the accuracy of the results was verified using the remaining 50%. Verification of the two results showed 95.03% and 95.19% high accuracy, respectively, as shown in Figure 12. This reflects the fact that both results detected almost all of the oil spill areas and the numbers of biogenic film pixels were relatively low in comparison. The results confirm that using the proposed algorithm, the ocean surface is less likely to be classified as oil and the removal effect of biogenic film is improved. However, the results would not be enough to present the robustness of the proposed method. To overcome this limitation, further validation process should be performed by using several oil spill accidents including different wind situations and look-alikes.

## 5. Conclusions

The most important element if the research of oil spill detection using radar images is to distinguish look-alikes from actual oil spills. Some look-alikes can be identified using ancillary data such as currents and winds, but some look-alikes are still difficult to distinguish from oil spills, affecting the accuracy of detection. To solve this problem, research has been carried out to consider morphological features using object-based ANN. However, as mentioned earlier, the accuracy of the method is significantly reduced except when the oil spill has a distinctive strip shape and in the case of large oil spill accidents, it is difficult to divide the dark spot on an object-by-object.

In this study, we applied the pixel-based ANN to solve this problem. However, result of pixel-based ANN is highly dependence on the input neurons. So, we used intensity image and phase information to detect the oil spill in pixel unit with high accuracy. In addition, in order to generate for accurate probability map, we also designed a preprocessing step to create an input layer optimized for oil spill detection; by eliminating false-detective and un-detective factor in each layer and by enhancing the contrast between the background pixel and the target pixel. In this study, an oil spill probability map was constructed by applying ANN with input neurons, including an NL-means filtered intensity map, a normalized texture map of intensity, and a coherence and phase texture map. The proposed algorithm shows improved results and its accuracy of about 95.19% is validated by using an ROC curve. The results of comparing the two probability maps according to the presence or absence of phase information, it can be seen that the probability of oil spill detection is increased when the phase information is used. Although it is slight, the probability of the oil-free surface being classified as an oil spill is entirely reduced. That is, the phase information enhances the accuracy of the classification by strengthening the contrast between the pixel in which the oil exists and the pixel that does not exist. It is also confirmed that by using the phase information together, it reduces the probability of about 0.3 in the look-alike with weak damping effect.

As a result, in this study, each image used for oil spill detection was optimized to produce a high accuracy probability from the pixel-based ANN. Its results show that the probability of detecting look-alike is significantly reduced while ensuring the detection of oil spills. In addition, by reducing the factors that cause false detection in pixel units, the detection was successful even when the morphological characteristics of the oil were not clearly revealed, such as the case of the large oil spill accident. Thus, it is expected that the accuracy of oil spill detection can be dramatically increased with additional data, which could remove look-alike regions such as low-wind zone and rainy cell.

The proposed algorithm should be checked using other images that include both oil spills and look-alike with weak damping effect. At present, the image used in this study is most appropriate the condition but if more image which includes oil spill area and look-alike is acquired by using co-polarized dual-pol mode, additional experiments using the image will be required. In addition, the current algorithm uses a median filter to remove the ship but it causes loss the detail of the image, so it will be more effective to detect the ship using the ship detection algorithm and lower the probability of the corresponding pixel in the modified algorithm. It is also necessary to further study what reduces the noise level of the texture map of the intensity image effectively.

## Figures and Tables

**Table 1:** Characteristics of TerraSAR-X Used in this study.

Quantity	Value
Acquisition date	16 November 2007
Imaging mode	Strip map
Orbit	Descending
Incidence angle (deg.)	25.85
Polarization	Dual-pol. (HH + VV)
